# The impact of chronic kidney disease on patient and caregiver quality of life: A qualitative study in Spain

**DOI:** 10.1371/journal.pone.0341371

**Published:** 2026-03-16

**Authors:** Nuria Aresté-Fosalba, Marta Cobo Marcos, Alfonso Segarra Medrano, Alberto Ortiz Arduán, Daniel Gallego Zurro, Juan Carlos Julian Mauro, Fernando Gutiérrez Nicolás, Raül Rubio Renau, Maie Khalil, Carlota Solà Marsiñach, Simona Gradari, Unai Aranda, Miren Sequera Mutiozabal

**Affiliations:** 1 Department of Nephrology, Virgen Macarena University Hospital, Seville, Spain; 2 Department of Cardiology, Hospital Universitario Puerta de Hierro Majadahonda (IDIPHISA), Madrid, Spain; 3 Centro de Investigación Biomédica en Red en Enfermedades Cardiovasculares (CIBERCV), Madrid, Spain; 4 Servicio de Nefrología, Hospital Universitario Arnau de Vilanova, Lleida, Spain; 5 Department of Nephrology and Hypertension, IIS-Fundación Jiménez Díaz UAM, Madrid, Spain; 6 RICORS2040, Madrid, Spain; 7 Departamento de Medicina, Facultad de Medicina, Universidad Autónoma de Madrid, Madrid, Spain; 8 Federación Nacional Alcer, Madrid, Spain; 9 Unidad de Investigación del Hospital Universitario de Canarias, Canarias, Spain; 10 Evidence Generation Department, A Piece of Pie, Barcelona, Spain; 11 Medical Department, AstraZeneca Spain, Madrid, Spain; Monash University, AUSTRALIA

## Abstract

Patients’ quality of life is a key consideration of chronic kidney disease care. Nonetheless, qualitative research on patients’ experience, quality of life, and frequent associated comorbidities remains limited. This study identifies aspects of the disease experience, healthcare journey, and caregiver experience contributing to quality of life for early-stage (stage 3 CKD) and advanced chronic kidney disease (stages 4–5) patients and their caregivers. In this cross-sectional, observational, multicenter study based on qualitative methodology, participants completed a general (Short Form-36) and disease-specific (Kidney Disease Quality of Life Short Form−36) quality of life assessment and semi-structured interviews. Quota and convenience sampling were used to enroll 36 patients selected by clinicians based on chronic kidney disease stage and the presence of key comorbidities (heart failure, hyperkalemia). Twelve caregivers were also invited to participate in patient interviews. All data were examined thematically. Three themes emerged: (1) Impact of chronic kidney disease on quality of life throughout the patient journey (physical, social, and emotional); (2) attitudes toward the disease (characterized by ‘acceptance’ or ‘powerlessness’); and (3) caregivers’ role and burden. Patients’ characteristics and caregiver support must be considered for designing medical interventions and improvement of patient-doctor communication. Family caregivers are pillars clinicians should rely on to raise awareness about chronic kidney disease’s relevance among patients and families.

## Introduction

Chronic Kidney Disease (CKD) currently affects more than 10–15% of the Spanish population, [1] being the second fastest growing cause of death between 2006–2016.[2] Estimates indicate that the total number of patients on kidney replacement therapy will have doubled by 2030.[3] Furthermore, CKD prevalence is higher in people with cardiovascular risk factors, and is often associated with other conditions, complicating its management.[4–6] Overall, the high prevalence, morbidity, and mortality rates, as well as the costs associated with diagnosis and management, make CKD a public health challenge, both in Spain and worldwide. As documented in the literature, quality of life (QoL) can help to predict CKD progression to kidney failure, dialysis, and death.[7,8] Thus, improving QoL has the potential to make a significant contribution, reducing morbidity, mortality, and associated costs. In this respect, several studies have identified predictors of QoL at different stages of CKD, such as hemoglobin level, glomerular filtration rate (GFR), hospital admissions due to comorbidities, education, age, and gender.[9–11] Other studies have assessed the impact of treatment and patient-associated factors on QoL. For instance, patients undergoing peritoneal dialysis (PD) at home report better QoL than those undergoing hemodialysis (HD) in a clinical setting.[12,13] Depressive symptoms and pain in patients receiving HD are independently associated with impaired QoL, [14] and their psychosocial distress is associated with increased hospitalization and more rapid kidney function deterioration.[7,15,16]. Perception of social support is associated with medication adherence– however–both can be attenuated in case of depression.[17] More generally, CKD and associated treatments represent an important burden on people’s lives, making everyday life more complex, due to the unpredictable nature of the disease.[18] For this reason, in the field of clinical trials, SONG-CKD (Standardized Outcomes in Nephrology Chronic Kidney Disease) protocols are being implemented to establish a consensus between patients and healthcare professionals regarding the reporting of outcomes, aiming to promote patient-centered care and improving patients’ experience.[19,20] In terms of measuring changes in QoL over time, studies showed a decrease in the early stages, as well as increased impairment of the physical domains compared to the mental as time went on. This could be attributed to psychological adaptation to the chronicity of the disease.[9]

As highlighted by several qualitative studies, the management and treatment of the disease is highly impacted by structural and relational factors.[21] Thus, social, cultural, and other factors not included in PRO questionnaires may affect QoL. Factors such as adaptation to illness and treatment, social support, and satisfaction with medical staff affect QoL assessment.[22] Therefore, understanding the patient’s social context, perceptions and psychological conditions is essential to obtain a more accurate and holistic picture of the disease burden’s impact on patients’ and their caregivers’ QoL.

The aim of this qualitative study was to identify aspects of the disease experience and healthcare journey important to patients and caregivers that could add complementary depth and context to measuring QoL. The themes could be tested as factors in future quantitative studies, discerning why associations may exist between variables. More specifically, this study focused on identifying what was most important to patients with early-stage CKD (stage 3), and advanced CKD (stages 4–5), and their caregivers concerning their QoL, disease experience, and healthcare journey in Spain.

## Materials and methods

### Study design

This was a cross-sectional, observational and multicenter study based on qualitative methodology carried out with 36 patients and 12 caregivers. Qualitative methodology allows an in-depth generation of knowledge about patients’ perspectives concerning their priorities and beliefs towards their condition.[23] Nevertheless, qualitative studies in nephrology are scarce.[24,25]

Participating patients had a diagnosis of chronic kidney disease (between stages 3–5) and were recruited in the nephrology and cardiology departments of three Spanish hospitals to ensure geographic representation: (kept anonymous). After obtaining written informed consent from patients, doctors compiled information related to patients’ personal characteristics, disease and treatment, and shared their contact information with two ethnographers. The ethnographers then contacted the patients by either encrypted instant messaging tools or by phone call. On twelve occasions the interview was also conducted with a caregiver; in those cases, written informed consent was given by both patient and caregiver beforehand. Open-ended interview questions were used to gain insight into the impact of chronic kidney disease on the quality of life of patients and their caregivers.

### Sample and recruitment

Patients and caregivers were recruited using consecutive sampling. Recruitment started on the 15^th^ of November of 2021 and ended on the 31^st^ of August of 2022. Patients ≥18 years old with a clinical diagnosis of CKD were eligible. The patient sample was stratified by 17 patients with a documented diagnosis of Heart Failure (HF) with reduced ejection fraction (HFrEF) according to ESC guidelines and 21 patients with a documented diagnosis of hyperkalemia (HK), documented serum potassium >5.5 mEq/L or documented ER visit for a HK episode during the previous control visit or before. The inclusion criteria were chosen given that HF and HK were identified by the study’s scientific committee and existing medical literature as the main comorbidities of CKD and, therefore, as having significant impact on QoL.[26–29] Some patients had both HF and HK, while some had none. Patients were excluded if they were hospitalized at inclusion or had a cognitive impairment. Caregivers were defined as people who accompanied the patient throughout the patient journey. There were no exclusion criteria for caregivers.

### Data collection and analysis

Patients participated in a single 90-minute remote semi-structured interview via phone call or video call. Remote interviews avoided fear of COVID-19 contagion. The 12 caregivers included in the study were asked to join for the entirety of the patients’ 90-minute interviews. The three female ethnographers had PhDs and a master’s degree in the social sciences and an average of 7 years of experience conducting fieldwork. They were employed as social scientists at the time of the study. The semi-structured nature of the interviews ensured that all known areas of interest were covered and left the opportunity for patients to raise and discuss any issues of concern. The information obtained from caregivers was used to compare and add to the view of the patients. Interview transcripts and fieldwork notes were analyzed thematically by two ethnographers. Each ethnographer independently coded all fieldwork materials. Codes were then compared and organized by themes in a coding tree arranged according to QoL dimensions and disease stage.[30–32]

Themes were identified by topics emerging directly from the data (inductive inference) and applying prior knowledge (abductive inference).[33] All selected themes had reached empirical saturation, the point at which new data no longer emerge.[34]

Additionally, patients completed two patient reported outcome measures (PROMs) at the beginning of the interviews in the unassisted presence of the ethnographer. These were the generic SF-36 and the disease-specific Kidney Disease Quality of Life Short Form 36 (KDQoL-36) questionnaires.[35,36] PROMs were only used as a conversation starter, instead of as a quantitative analysis tool. This strategy was useful to approach sensitive topics, to prompt patients to self-reflect on their condition without the intervention of the ethnographer, and to shed light on the dynamics between patients and caregivers.

Finally, descriptive statistics such as count and percentages were exclusively used to present a clearer view of qualitative variables related to patients’ clinical and sociodemographic characteristics.

### Ethics statement

Before patient recruitment, all study materials were reviewed and approved by the Research Ethics Committee of the (kept anonymous) on the 30^th^ of September of 2021. The study was given the following code: (kept anonymous). Written informed consent was given by participants before being enrolled in the study. Before the interview, patients and caregivers were informed of the research objective and interviewer’s affiliations and asked to verbally confirm their informed consent and permission for the interview to be recorded and transcribed. All data were pseudonymized to protect participants’ confidentiality. The study was conducted following the principles outlined in the revised version of the Declaration of Helsinki, Good Clinical Practices (GCPs). COREQ Guidelines were followed.

## Results

Among the thirty-six participant patients, the mean age was 70 years (range: 42‒84 years), of which 30% were women. The average time of diagnosis of CKD was 6.7 years. 28% of patients were at stage 3, 28% were at stage 4, and 44% were at stage 5. Of the latter, 50% were on pre-dialysis and 50% on dialysis. Of those patients who were on dialysis, 50% were on peritoneal dialysis while 50% were on hemodialysis. Four patients were on a waiting list for a kidney transplant, and two patients had already had a transplant. Median time on dialysis since diagnosis or kidney transplant was five years. Median time on dialysis before transplant was three years. Table 1 summarizes the clinical and demographic characteristics of the patients.

**Table 1:** Patient characteristics (N = 36).

Gender, women, n (%)	11 (30)
Age, mean (range), years	70 (42-84)
Age category, n (%), years	
< 60	7 (19,5)
60–69	7 (19,5)
70–79	13 (36)
≥ 80	9 (25)
CKD stage, n (%)	
3	10 (28)
4	10 (28)
5, of which	16 (44)
5 Pre-dialysis	8 (22)
5 On dialysis	8 (22)
Peritoneal dialysis	4 (11)
Hemodialysis	4 (11)
Time since diagnosis, median, years	6.7
Comorbidities, n (%)	
Heart Failure	17 (47)
Hyperkalemia	21 (58)
Diabetes Mellitus Type 2*	9 (32)

(*) Reported spontaneously by patients.

Data expressed as n (%) unless indicated otherwise.

For the 12 family caregivers, 78% were women. Two caregivers (17%) were children (one male and one female) and the rest (83%) were spouses (all female). Mean age of caregivers was 67 years (range: 49–83 years). There was no instance of assisted peritoneal dialysis.

The qualitative analysis of the semi-structured interviews, crossed-checked with the two PRO questionnaires, identified three themes related to QoL with CKD: 1) Impact on QoL throughout the patient journey; 2) Distinctive attitudes towards the disease; and 3) Caregivers’ role and burden.

### Theme 1. Impact of CKD on QoL throughout the patient journey

Patients identified four key stages in their patient journey with CKD: 1) CKD diagnosis; 2) stages 3–4; 3) pre-dialysis; and 4) dialysis. CKD was found to impact patients’ QoL in different ways in each of these stages.

#### CKD diagnosis.

The CKD diagnosis was generally communicated after a routine blood test for other comorbidities. Most frequently, patients were not expecting a CKD diagnosis. Diagnosis had a much greater impact on those patients who were in advanced stages and to whom the need for dialysis in the near future was mentioned. Diagnosis did not have a great impact on patients who were in stages 3 and 4 and had other comorbidities such as HF or diabetes. Furthermore, diagnosis had a significant impact on those who considered themselves as “healthy” and with fewer comorbidities.

#### Stages 3–4.

Many patients in stages 3 and 4 frequently reported not experiencing any symptoms associated with kidney function, describing their disease as silent. Many patients feared the implications of CKD progression, while others did not give it too much importance. The latter was particularly salient in those patients with HF or other comorbidities such as diabetes, who perceived them as their main health problem and CKD as its subproduct. Older patients sometimes perceived CKD symptoms as a result of the aging process.

For these patients, the main physical burden of living with CKD was dietary and fluid restrictions. We identified a gender bias in this aspect. Generally, male patients were burdened by not being able to enjoy the foods they wanted. For some, this had a negative impact on their relationship with their wife as their caregiver. It also had an impact on their social life, because in order to avoid ingesting prohibited foods or drinks they avoided going out to reduce the temptation. In addition to this, female patients were burdened by having to adapt their cooking to fulfill their new dietary requirements. Furthermore, patients with HK were not aware they suffered from HK, and therefore did not attribute dietary restrictions to HK but to CKD.

#### Pre-dialysis.

Among patients on dialysis, this stage of the patient journey could be subdivided into three key moments: 1) the medical consultation during which the need to undertake dialysis in the near future was announced to the patient; 2) the session with a nurse where dialysis options and mechanisms were explained; and 3) the time in between.

The medical visit where the need for dialysis was announced engendered feelings of fear, helplessness, and being completely lost. It was described as a very difficult moment by most patients. Due to their previous lack of awareness about CKD severity and progression, many were shocked at the news of dialysis, and were angry and resentful with their doctors for not having warned them. Patients reported fear of physical limitations and disability, which negatively impacted QoL.

These feelings and their impact on QoL remained present until the time the dialysis information consultation was held. During that session, a nurse explained to patients and caregivers the option to undergo peritoneal dialysis, explaining all relevant procedures. Patients expressed feeling relieved after this consultation; many did not know the option to undergo peritoneal dialysis from home existed and found this option much less dreadful than hemodialysis. Additionally, patients felt taken care of by the nurse undertaking the consultation; their understanding of what they would need to do was enhanced, which made them feel much more in control of the situation. As a result, they felt grateful to the nurse undertaking the consultation. That moment considerably improved their QoL.

#### Dialysis.

Dialysis had a major impact on patients’ QoL. Several patients on peritoneal dialysis experienced sleeping problems due to being connected to the machine during the night, especially during the first weeks of dialysis. Furthermore, patients reported concerns about self-image due to having a ‘tube sticking out’ of their body. For some, this translated into problems in sexual functioning. Additionally, patients saw their social life reduced. Having to be connected to the dialysis machine reduced travel possibilities, especially for those undergoing hemodialysis, and generally complicated making plans with others. As in stages 3 and 4, dietary limitations reduced the number of social outings where food and drinks were involved, to avoid falling into the temptation of consuming prohibited foods. Patients reported a lack of understanding of the limitations imposed by CKD in the social context, which contributed to the reduction of their social life. Additionally, patients feared having to depend on loved ones and becoming a burden to them. This fear was associated with the routines related to dialysis, such as transportation to and from the hemodialysis centers, and the need to clean and prepare the machine in case of peritoneal dialysis.

Throughout this stage of the patient journey, patients manifested hope for a kidney transplant and the consequent possibility of recovery. According to patients, this hope had a positive impact on their QoL.

Table 2 presents quotes on the impact of CKD on QoL throughout the patient journey.

**Table 2:** Quotes on the impact of CKD on patients’ QoL throughout the patient journey.

Journey stage	Illustrative quote (gender and age in years)	Group	CKD Stage
**Diagnosis**	“It was in 2012, but I didn’t feel anything, I don’t remember any symptoms or anything bothering me. I had had some cysts in my kidney, but it was a long time ago, and sometimes my back hurt, but nothing else.” (Male, 70)	HK	4
	“I think the heart has caused the kidney damage, because the disease itself has been slow and progressive, I don’t even remember the year of diagnosis, but it was detected in blood tests, my creatinine was very high.” (Male, 82)	HF, HK	4
	‘When I got the diagnosis, it was like a splash of cold water... I was very surprised, and I was, I still am, very afraid... The doctor was very curt; I asked her how was I going to work and she replied that ‘anyway I was no longer working (because I had a heart problem)... She had very little empathy. (Female, 53)	HK	4
	‘When the nephrologist diagnosed me with CKD, I was very distressed; I feared I would have to go on dialysis. (Male, 83)	HK	3
	‘When he saw the results of the analysis the nephrologist told me: “this is the last door before going to dialysis” and I was very surprised. Now looking back, I wish that doctors would have given me tools, together with the diagnosis, so that I could have been psychologically treated. (Male, 47)	HK	5 HD
**Stages 3** **and 4**	“Now I am stable, but if the kidney progresses, we think we will need help at home, I do not know how we will manage the dialysis or how I will be in the near future” (Female, 84)	HF	4
	“What worries me most is not my current state with CKD, because I don’t notice anything, any symptoms, but the future. I don’t know how I’m going to evolve, and the truth is that the very idea of dialysis scares me” (Male, 83)	HK	3
	“What’s most difficult for me to deal with are the food restrictions, not being able to eat everything I want. I don’t deprive myself of a glass of wine some weekend, but the food thing is what I have most difficulty accepting.” (Female, 63)	HK	3
	“The transplant would be ideal, we would end the problem partially or totally, I would not be restricted by the disease” (Male, 68)	HK	4
**Pre-dialysis**	“I was very scared when they told me I would have to do dialysis. Quality of life means moving, going to the vegetable garden or the village, and not having to go to the hospital 3 times a week for dialysis. But when the nurse explained to us that we could do the dialysis at home, I felt much better. I’m so grateful to that nurse, he was an angel.” (Male, 78)	–	5 (pre-dialysis)
**Dialysis**	“The hardest thing is to go on hemodialysis... I take it as if I have to go to work and work 15 hours a week. It’s very difficult for me because it limits me a lot, I can’t go on vacation, I can’t continue working, I can’t travel like I did before.” (Male, 61)	HK	5 HD
	“I feel like a burden to my family, yes. More and more I depend on my wife and that bothers me. I’m not as independent as I used to be” (Male, 61)	HF, HK	5 PD
	“You lose almost all your social life, because I don’t go to the bar to have a beer with friends, I can’t go to romerias to not get too thirsty. My friends don’t understand that I can’t drink and that’s why I stay at home with my wife” (Male, 73)	HF	5 PD

Abbreviations: CKD, chronic kidney disease; HD, hemodialysis, PD; peritoneal dialysis; HK, hyperkalemia; HF, heart failure.

### Theme 2. Distinctive attitudes toward stage 5 CKD and their impact on QoL

Among patients with CKD stage 5, we found two contrasting sets of narratives informing two distinct attitudes: acceptance and powerlessness. According to patients, these attitudes influenced the impact of CKD on QoL.

When acceptance was the main characteristic of patients’ attitudes, they:

a) expressed optimistic feelings
b) focused on what they could do and created routines beyond treatment;
c) found comfort in feeling accompanied by their relatives, and sought to surround themselves with their family;
d) tried to view the security of hospital-administered dialysis in a positive manner, seeking to relieve domestic pressures of dialysis such as cleaning or maintenance; and
e) sought autonomy by relying heavily on family support to take dialysis at home.

When powerlessness was the main characteristic of patients’ attitudes, they:

a) expressed feelings related to discouragement and depression;
b) would like to give up and not rely on kidney replacement treatment anymore;
c) felt more isolated and lonelier from dialysis routines; and
d) regardless of the type of dialysis they took, felt hopeless, socially isolated, and feared overloading their caregiver.

Examples of patients’ attitudes towards stage 5 CKD can be found in Table 3.

**Table 3:** Examples of patients’ attitudes towards stage 5 CKD.

Attitudes	Illustrative quote	Group	CKD Stage
**Acceptance**	“Your mind goes to bad places, since I have a lot of time to think during dialysis, I wonder why this has happened to me? Even so, I quickly try to think about something else and remember the good times in life, I mentally go to the mountain, to places where I have found peace being alone. Memories bring me back joy.” (Male, 73)	–	5 PD
	“I feel satisfied with what I have achieved in my life, with my family and with my grandchildren... I try to concentrate on what I like which is painting and reading about Madrid” (Male, 82)	–	5 pre-dialysis
	“I will be able to go to the hospital on alternate days, this will allow me to go to the beach, have the next day off to rest. After all, it will help me get out of the house and distract myself.” (Male, 64)	–	5 pre-dialysis
	“I’m not worried about my health. You don’t realize you have kidney disease, and when I have a problem, I face it with good attitude. I get sad when I think about family.” (Male, 78)	–	5 pre-dialysis
	“Yes, I am physically very limited, very sick, I will not hide it. You have seen how I have checked the boxes on those questionnaires. But I try to stay positive and look for a reason to keep going.” (Male, 61)	HK	5 HD
**Powerlessness**	“I received the news of dialysis very badly because it is a different quality of life. I asked the doctor if I could quit, I’d rather die, but then, of course, it’s not so easy... I have my children, my grandchildren, things to see. I know it’s a life solution, but at first, I had a really hard time. Now seeing what it is, I take it with resignation.” (Female, 79)	–	5 PD
	“Look... I’ll say it, just as the machine has given me life... otherwise the cancer here is decontextualised, it has also taken it from me.” (Male, 61)	HK	5 HD
	“In three words I would say that I feel stunned, depressed and scared about dialysis... I will have very little quality of life... Being plugged into a machine the rest of my days is hard to take on... I would have liked to have been told more about the disease, I did not imagine that ending like this was an option” (Male, 78)	–	5 pre-dialysis
	“I wouldn’t do anything, if it were up to me, I would let myself go, I’m not afraid of death. I was told I can do dialysis at home and my son insisted on it. I do it so as not to leave my wife with a pension that would not be enough for her at all. But well, although I have some doubts with that dialysis, I have accepted it grudgingly” (Male, 82)	HF	4

Abbreviations: CKD, chronic kidney disease; HD, hemodialysis, PD; peritoneal dialysis; HK, hyperkalemia; HF, heart failure.

### Theme 3. Caregivers’ role and burden

Most of the caregivers who were interviewed along with CKD patients were family members (spouses or children) who were not paid for their services and were therefore designated as informal caregivers. Seventy-eight percent were female. Our study found that CKD also had a considerable impact on their lives.

First, CKD had a negative impact on the emotional wellbeing of caregivers, especially the medical visit where patients were told about impending dialysis. They described feelings of fear and helplessness. They worried about the health of the patient and were uncertain about the implications of dialysis, both for the patient and for themselves. Due to their worry about the added burden of peritoneal dialysis at home, some caregivers refused it and pressured the patient to undertake hemodialysis at the hospital. Like patients, caregivers expressed relief after they obtained clarity on the possibilities and implications of dialysis during the pre-dialysis consultation with nurses.

Second, CKD had a negative impact on the social life of many caregivers, which was restricted due to not wanting to leave the patient alone. This was felt particularly when caregivers were spouses.

Third, caregivers were burdened by the time spent and the physical labor required to care for CKD patients. Caregivers were often responsible for the preparation of patients’ low sodium or potassium diets. Additionally, caregivers helped patients carry heavy objects, such as shopping bags, and frequently drove patients to medical appointments. Caregivers were also in charge of the maintenance of the peritoneal dialysis machine. When caregivers were the adult children of the patient, they were left to take on all health-related decision-making, especially if the patient was a widow or widower. We identified a gender bias in the provision of care and therefore its impact. Male caregivers tended to more frequently assume tasks such as carrying heavy objects and driving the patient to medical appointments. Female caregivers tended to assume to a greater extent tasks related to the preparation of a low sodium or potassium diet and the maintenance of the peritoneal dialysis machine. As a result of traditional gender roles in Spain, male patients tended to depend on their wives’ care to a greater extent than vice versa. Consequently, female caregivers who were spouses reported feeling alone and overloaded by care-related tasks, to a much greater extent than male caregivers.

Examples of patients’ and caregivers’ responses related to “Caregivers’ role and burden” can be found in Table 4.

**Table 4:** Examples of the caregivers’ role and burden.

Caregivers’ role in CKD	Group	CKD Stage
“My son is the one in contact with all the doctors. He takes care of the appointments and comes to see me almost every day. I’m doing what he tells me.” (Female, 75)	HF, HK	4
“My son is like if my husband existed, but with more energy. He takes care of everything, the house, the doctors, dialysis, everything... Of course it makes me feel good because I see him doing it with pleasure, but I also see that he is exhausted, he does not rest. Nobody can stand that, and I fear he will get sick.” (Female, 79)	HF	5 HD
“I keep a tighter control, the doctor visits, the shopping, the meals. My husband takes little care of his health, and sometimes I think that, if I miss one day, he will not know how to fend for himself... That’s why I don’t want the dialysis machine at home, I wouldn’t know what to do with it or how to take care of something else.” (Caregiver of male, 78)	–	4
“My wife is my nurse: she chases me with the bottle of water to drink, she takes care of the food, she watches what the doctor says. She has always been dedicated to her chores and to me” (Male, 82)	HF, HK	4
“It is my wife who is in charge of the food, of cooking as we have been told, without potassium” (Male, 83)	HK	3
“He gets along well except for the issue of unsalted food and having a long list of forbidden foods. I take care of the meals. Some have to be soaked, washed and boiled several times, I don’t mind the extra work... anything so he doesn’t have to live tethered to a machine.” (Caregiver of male, 70)	HK	4

Abbreviations: CKD, chronic kidney disease; HD, hemodialysis, PD; peritoneal dialysis; HK, hyperkalemia; HF, heart failure.

## Discussion

This study, which aimed to identify the impact of CKD on QoL for patients with stage 3–5 early- and advanced-stage CKD and their caregivers, generated three main themes: 1) Impact on QoL throughout the patient journey; 2) Distinctive attitudes towards the disease; and 3) Caregivers’ role and burden. These themes should be considered in future studies as factors to improve the QoL and care of CKD patients.

In Theme 1 (Impact on QoL throughout the patient journey), we documented patients’ perceptions and QoL impact in stages 3–4 - early to mid-stage of CKD-, a period for which literature is scarce, since most studies focus on stage 5 late-stage CKD and kidney failure. Our finding that the diagnosis had a greater impact on people who considered themselves “healthy” and with fewer comorbidities complements the interpretation of previous studies reporting that earlier-stage patients had a lower perception of the disease’s consequences.[10,37,38] As patients journey through the stages of CKD and healthcare milestones, their perception evolves in a dynamic manner. For example, when they are given the news they must receive dialysis, their perception of the treatment in the period before and after the nurse pre-dialysis education session shifts from fearful to more relieved, improving patient QoL. This evolution occurs in parallel with their social role as well, as seen in a Spanish qualitative study that showed that CKD’s dietary restrictions and pharmacological treatments burden women more. This is confirmed by our finding that female patients who had to act as their own caregiver to adapt their diet resulted in extra burden.[39] These results may be interpreted as a result of women’s socialized role as caretakers of their family.[37]

It is apparent that the negative impact of feeling restricted dietarily on QoL found in early stages may be reflective of the fact that patients do not feel symptoms of the disease initially. Later, in stage 5 dialysis, complaints about diet are less expressed. The focus and importance given to these dietary restrictions needs to be understood in light of patients’ lack of awareness of the severity of CKD and its future complications. This phenomenon is comparable to the analogy of cancer patients who rarely ever mention diet as impacting their QoL, as it pales in comparison to their fear of the condition itself and its treatment. For this reason, the use of quantitative PRO questionnaires became an important tool to initiate the conversation about QoL during the interview. This method enabled researchers to explore the ‘why’ behind the ‘what’ and to distinguish between different channels to provide information.

In Theme 2 (Distinctive attitudes towards the disease among stage 5 patients), our finding that patients worried about being a burden to their caregivers and being unable to support their families aligns with a Spanish qualitative study which showed that convergence of a patient’s self-identity with the perceptions of dialysis generated a protective role to avoid feeling burdensome.[39] This finding has implications regarding the importance of QoL as a predictive marker for studies demonstrating that negative perception of social/family support is associated with poorer health outcomes.[40–42] Furthermore, patients with the attitude of powerlessness reported a higher impact on the mental aspect of QoL. This points to the importance of prioritizing psychological interventions for this segment.

Furthermore, the present data may explain why providing patient education may not be enough to ensure patients understand their condition. For example, a Spanish study showed that a lack of knowledge persisted throughout the journey despite the educational information offered to patients.[39] Our findings could contribute to the explanation of these results, since the lack of psychological assistance during disease progression negatively impacted patients’ and caregivers’ QoL, likely precluding effective patient education. In this regard, our findings suggest that when patients show an attitude characterized by powerlessness, providing educational information is insufficient. In such cases, psychological interventions may be more effective to ensure that patients understand their condition.

Our findings of the distinct attitudes and key moments of the journey impacting QoL may reflect patient acceptance of the situation and optimism for transplantation. These perceptions could be used to inform a novel patient categorization, complementary to biomarkers, to provide new associations with clinical outcomes.[43] Addressing the need for education and social support may help to increase treatment adherence, minimize the impact of exacerbations, and modify health-related behaviors, leading to better outcomes. Furthermore, raising awareness of CKD and changing its perception can encourage proactive behaviors.

In Theme 3 (Caregivers’ role and burden), our finding that caregivers pushed for the choice of hemodialysis to avoid the additional home burden of peritoneal dialysis aligns with a meta-analysis that found family carers censored treatment option information incompatible with their own wishes.[47] The divergence of caregivers’ dialysis perception from that of patients’ points to the importance of measuring QoL from multiple perspectives. Further, the gendered strain of dietary limitations on the marital relationship between a male patient and their spouse caregiver highlights the importance of context, as a formal caregiver would be less likely to engender this effect on QoL.

A randomized study of a caregiver-based educational program for patients synchronously undergoing hemodialysis showed that caregiver QoL significantly increased after the intervention.[48] Another randomized study notably found that caregiver-centered education (implemented during HD) reduced the care burden more effectively than patient-centered education (implemented prior to HD initiation).[49] This finding emphasizes the importance of directly educating caregivers. Considering this, our finding that patient and caregiver QoL was negatively impacted during the pre-dialysis stage and improved according to patients after the nurse-lead pre-dialysis education session fosters a novel suggestion: to minimize the time between the medical consultation outlining the need for dialysis and the pre-dialysis educational session. Providing joint patient-caregiver and individual education sessions before initiation could not only reduce care burden and improve QoL, but also impact the choice of dialysis type. For this reason, it is important to continue designing studies that separate the elicitation of caregiver views from that of patient views.

Regarding our finding that caregivers were concerned about social and health deterioration, female caregivers reported being lonelier and more overburdened. This occurred whilst taking care of generally male patients (typically their partner). Divergences between caregivers’ and patients’ perceptions and attitudes toward the disease are relevant for female patients who have male caregivers, as this may manifest as an underestimation of and inattention to female patients’ condition. This underestimation of not taking the condition as seriously or not paying as strict attention to treatment and lifestyle changes may lead to lower QoL and worse patient outcomes.[44–46] These observations justify the integration of caregivers’ perspectives into future quantitative and qualitative studies to improve outcomes.

### Strengths and limitations

Strengths of this study include the ethnographic approach, which involved in-depth interviews with patients at their homes and with their caregivers; this allowed the collection of relevant data not normally shared in healthcare facilities. Both patients and caregivers could speak freely and explain the aspects of the disease that they considered important. However, the first limitation of this study is that the small sample size, which - although not unusual in qualitative research which requires extensive and detailed analysis of each patient - may not fully represent the diversity of people with CKD in Spain. Secondly, as is the case in other ethnographic studies, interviewing patients and caregivers together may have inadvertently resulted in individual perspectives being altered or withheld. In future studies, it would be ideal to recruit more adult children caregivers as compared to spouse caregivers. It would have also been ideal to go beyond retrospective data by conducting two interviews, for instance, one held pre- and one held post-consultation with the nurse about pre-dialysis education, so as to measure the change in QoL. Finally, the consecutive recruitment method resulted in the enrolment of a mostly elderly CKD population. Therefore, the experiences reported in this paper may differ from those of a younger cohort; for instance, the themes described in this paper do not reveal worries about parenthood, educational disruption or career opportunities, which have been found to be major concerns of young adults with CKD [50–53].

## Conclusion

In conclusion, the impact of symptoms and treatment burden on QoL is significant in individuals across the CKD spectrum. This finding reinforces the relevance of qualitative research which uncovered the following: (i) the need to develop accurate, reliable patient-centered initiatives for more holistic care that can detect changes over time in interventional settings; and (ii) improve patient-doctor communication and attention to patient-important outcomes. With this, progress will be made in understanding and applying insights from the experience of living with CKD and its most frequent comorbidities.

## Supporting information

S1 Table Results of the KDQOL-36 questionnaire. (DOCX)

S2 Table Results of the SF-36 questionnaire. (DOCX)

S3 Table Interview guide. (DOCX)
