# Supplementary material for: The impact of chronic kidney disease on patient and caregiver quality of life: A qualitative study in Spain
Source: PLoS One. 2026 Mar 16;21(3):e0341371. doi: 10.1371/journal.pone.0341371 (PMC12991225; doi:10.1371/journal.pone.0341371)
Supplement: S1 Table — (DOCX) [file pone.0341371.s001.docx]

| **S1 Table. Results of the KDQOL-36 questionnaire** | | | | | |
| --- | --- | --- | --- | --- | --- |
| **CKD Stage** | **Patients**  (n=) | **<50 in Effects of Kidney Disease***  n (% within stage) | **<50 in Burden of Kidney Disease**  n (% within stage) | **<50 in Physical Composite**  n (% within stage) | **<50 in Mental Composite**  n (% within stage) |
| 3 | 10 | 1 (10%) | 2 (20%) | 6 (60%) | 2 (20%) |
| 4 | 10 | 0 (0%) | 5 (50%) | 10 (100%) | 5 (50%) |
| 5 pre-dialysis | 7 | 0 (0%) | 4 (57%) | 4 (57%) | 5 (71%) |
| 5 dialysis | 8 (4 HD, 4 PD) | 4 (50%) | 6 (75%) | 6 (75%) | 3 (38%) |

*Higher scores indicate better QoL.
