# Supplementary material for: The impact of chronic kidney disease on patient and caregiver quality of life: A qualitative study in Spain
Source: PLoS One. 2026 Mar 16;21(3):e0341371. doi: 10.1371/journal.pone.0341371 (PMC12991225; doi:10.1371/journal.pone.0341371)
Supplement: S2 Table — (DOCX) [file pone.0341371.s002.docx]

| **S2 Table. Results of the SF-36 questionnaire** | | | | | | | | | | |  |
| --- | --- | --- | --- | --- | --- | --- | --- | --- | --- | --- | --- |
| **CKD Stage** | **Patients**  (n=) | **Physical functioning***  Mean (SD) | **Role functioning**  Mean (SD) | **Emotional functioning**  Mean (SD) | **Energy/fatigue**  Mean (SD) | **Emotional well-being**  Mean (SD) | **Social functioning**  Mean (SD) | **Pain**  Mean (SD) | **General health**  Mean (SD) | **Health change**  Mean (SD) | |
| 3 | 10 | 73 (39) | 60 (50) | 80 (41) | 70 (27) | 90 (18) | 86 (25) | 79 (27) | 52 (34) | 68 (31) | |
| 4 | 10 | 39 (38) | 40 (50) | 60 (49) | 50 (28) | 71 (24) | 59 (35) | 56 (37) | 34 (32) | 53 (32) | |
| 5 pre-dialysis | 7 | 51 (44) | 46 (51) | 62 (50) | 39 (29) | 63 (38) | 43 (36) | 66 (38) | 41 (40) | 43 (24) | |
| 5 dialysis | 8 (4 HD, 4 PD) | 50 (44) | 25 (44) | 63 (48) | 46 (30) | 67 (28) | 63 (37) | 67 (33) | 35 (31) | 47 (41) | |

*Higher scores indicate better QoL.

SD=Standard Deviation
