# Supplementary material for: The impact of chronic kidney disease on patient and caregiver quality of life: A qualitative study in Spain
Source: PLoS One. 2026 Mar 16;21(3):e0341371. doi: 10.1371/journal.pone.0341371 (PMC12991225; doi:10.1371/journal.pone.0341371)
Supplement: S3 Table — (DOCX) [file pone.0341371.s003.docx]

| Ethnographic Guide - Patients  HYPERKALAEMIA AND CHRONIC KIDNEY DISEASE  DECEMBER 2020 |
| --- |

**
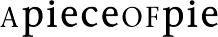
S3. Interview guide**

**Presentation**

Good morning/afternoon,

We'll start with some formalities, if that's okay.

First of all, thank you for participating in this interview. With your permission, we will record your responses to facilitate our internal analysis.

Please note that:

- Your answers are confidential and anonymous. Under no circumstances will your personal data be shared when the results are published.
- A Piece of Pie complies with all European and North American data protection laws and meets the guidelines set by the European Pharmaceutical Market Research Association (EphMRA) codes of conduct for market research.
- You may discontinue your participation in this investigation at any time and have the right not to answer any questions to which you do not wish to respond.

[MODERATOR: Start recording the videoconference.]

And now, I will introduce myself. My name is [XXX] and I work for A Piece of Pie, an innovation consultancy.

We are currently conducting a study on behalf of a pharmaceutical company, which would like to better understand the experience of patients suffering from hyperkalaemia and chronic kidney disease.

We would be very grateful if you could share your personal experiences with us and help us identify areas for improvement throughout your experience, from the time of the first symptoms to the current status.

Please note that there are no right or wrong answers, as we are interested in your opinions and personal experience.

Of course, your answers are confidential and anonymous; they will be addressed in an aggregated manner in our report.

***Reporting of adverse events***

A clinical study interview is about to begin.In compliance with the Royal Decree on Pharmacovigilance 577/2013, dated July 26, if during this meeting you report any adverse reactions, quality complaints, exposure during pregnancy, suspicions of infectious agent transmission, drug interactions, or special situations such as overdose, abuse, misuse, administration errors, medication errors, occupational exposure, or lack of efficacy related to the investigation of any sponsor's drug, this information will be collected and forwarded to the laboratory. In this case, do you authorize to be contacted by the sponsoring laboratory of the drug?

- Yes (ask for contact phone and write down): _______________________
- No

If you prefer not to provide your information, the report to Pharmacovigilance will still be made, but without disclosing your name or personal details. Alternatively, you may also choose to report it directly.

Note: This guide provides guidance during the interview to achieve the research objectives, the timing and sequence are adapted to the interviewee avoiding forcing answers.

**OBSERVATION GUIDE (during interview)**

*Observe the* ***interviewee during the videoconference.*** *How does he/she look? (For example, tired, relaxed, irritated, etc.) Is there anything that surprises you?*

*Focus on:*

- Dress.
- Accessories (e.g. watch, jewelry, wedding ring).
- Personal image (e.g. makeup, perfume, shaving, hairstyle).
- Body posture.
- Nonverbal cues (facial expression, comfort level with questions).
- Mismatch between verbal responses and body language.
- If applicable: interaction with healthcare professionals, hospital peers.
- If applicable: use of technological devices.

*Throughout the interview, when applicable, introduce observation about the environment and the participant into the conversation to encourage them to open up..*

**THE INTERVIEW (90 min)**

1. Meet the patient (15 min)
2. Meaning of quality of life (15 min)
3. Chronic kidney disease (15 minutes)
4. Life journey (15 min)
5. Assistance process (30 min)

[MODERATOR: Whenever you think the caregiver could provide additional information about any of the topics discussed, address the questions to him/her as well. If, on the other hand, you feel that it will be more valuable to ask certain questions only to the caregiver, do so after the patient interview. Always ask the patient for permission before talking to the caregiver. Tailor questions to how the patient refers to the condition and use the term provided by the patient to make the patient feel more comfortable.]

| **Area of exploration** | **1. MEET THE PATIENT (15 min)** |
| --- | --- |
| **Objectives and information** | - **Building a relationship with the patient** - **Better understand the person and their context** - **Understand their daily habits and routines** |
| **Ethnographic explanation** | *To begin, I would like to get to know you as a person and familiarize myself with your personal situation....* |

1. **Person:** I'd like you to introduce yourself and explain to me:
   1. How old is she? Who do you live with?
   2. Tell me a little more about yourself, what do you like to do? What are your hobbies? What do you do for a living? What do you value most now in your life? Why is that?
2. **Self-description:** If you had to describe yourself, what are the 3 words you would use? What comes to your mind when you think of yourself?
3. **External vision:** What do you think a good friend or relative would tell me if I asked him what [patient's name] is like? How do you think they would describe him?
4. **Values:** If you had to tell a young relative of yours what is the most important thing in life, what would you say? Do you have any kind of personal maxim? Why do you think it's important? [MODERATOR: If health issues arise, ask when it became important to have a healthy life in your family and/or social environment.]
5. **The patient's day to day:** Tell me what a “typical” day is like. What do you do from the time you get up until you go to sleep? Differentiate weekday with weekends (hobbies) [MODERATOR: inquire from the answers collected in the digital journal]
   1. What time of day would you say you like better? When do you feel more comfortable? Why is that?
   2. And what time do you like less? Why is that?
   3. What do you find most difficult to do?
   4. Is there anything he's had to give up lately? What do you miss?

| **Area of exploration** | **2. MEANING OF QUALITY OF LIFE (15 min)** |
| --- | --- |
| **Objectives and information** | - **Understand what is associated with quality of life** - **Understand the impact of different CKD symptoms on your quality of life** |
| **Ethnographic explanation** | *Now I'd like to talk to you about the things that you think about having a quality of life* |


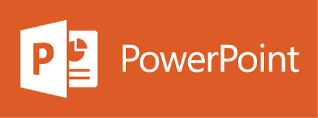


***EXERCISE #1: QUALITY OF LIFE***

Digital materials (see Annex):

- Quality of life map
- Quality of life images

Think for a moment about what it means to you today to have quality of life.

**[Moderator, please share screen or ask to open the pdf sent in advance to the patient].** You will see a series of pictures in the document. Please take a few minutes to look at them and tell me what they relate to your idea of what it means to have "quality of life." You can select the 3 or 4 images that best represent what quality of life means to you.


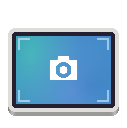
**[Moderator, place the images that are most relevant to the patient in the center of the map.** Once you're done, explain why you've chosen each of the images and placed them in each place. Also ask why you have discarded the rest of the images.

*Once the map is created, take a screenshot of the result*.

1. **Quality of life definition**: Now that you have completed this map, how would you define quality of life?
   1. What things allow you to have a quality of life? How do you feel about them?
   2. What other factors prevent you from having a better quality of life? How do you feel about them?
2. **Current feeling:** How do you feel at this point in your life? Do you have a little ritual that helps you especially in your day to day? [MODERATOR: Ask about eating habits, physical activity, cultural activities, etc.]
3. Is there anything in particular that bothers you? Could you give us an example?
4. What makes you happy?
5. If you could improve three things in your day-to-day life, which things would you choose? What would I give up?

| **Area of exploration** | **3. CHRONIC KIDNEY DISEASE (15 min)** |
| --- | --- |
| **Objectives and information** | - **Understanding the impact of CKD on the patient's life** - **Understand the patient's perception of CKD** |
| **Ethnographic explanation** | *Now I'd like you to talk to me specifically about the disease you suffer from...* |

1. **Chronic Kidney Disease**: I'd like you to explain to me in your own words how you see chronic kidney disease. [MODERATOR: If applicable, inquire from the answers collected in the digital journal]
2. **Explanation of the disease**: Do you remember how it was explained to you what CKD is? With what words? Who?
   1. Could it have gotten better? What did you miss?
3. **Living with CKD:** What is it like living with CKD? At what point do you feel you are becoming more “challenging”? What helps you overcome them?
4. **Other diagnoses:** Are you diagnosed with other diseases? Were they prior to CKD?
   1. Are they related in some sense?
5. **Impact on your quality of life**: [Moderator refer to quality of life exercise]. What images have you chosen that are impacted by CKD? Are any of the images we've discarded important?

| **Area of exploration** | **4. LIFE JOURNEY (15 min)** |
| --- | --- |
| **Objectives and information** | - **Understanding the impact of CKD on the patient's life** - **Understand the patient's perception of CKD** |
| **Ethnographic explanation** | *To continue I would like to briefly talk about the most important moments with the disease ...* |

1. **Significant moments with the disease:** Now I would like you to think about 5-6 significant moments since you were diagnosed with the disease. [Moderator: Record every moment. Pay attention to whether hospitalization, dialysis, and not to ask if you have experienced any of these moments spontaneously and if so, record them next to the other times].
   1. Why have you chosen precisely these moments?
   2. How did you live each of these moments?
   3. Which of them was the best for you? [Moderator: Ask what "best" means for this particular patient]
   4. Which one of them was the worst for you? [Moderator: Ask what "the worst" means and, if related to the patient's illness, ask] 🡪 Is there anything that could have helped them cope better with these particularly difficult times?
   5. Which of these moments changed your life to a greater extent? Why is that?
   6. **[For patients only]** What has been the role of your family/close environment since the diagnosis? What has changed in your relationship with them?
   7. **[Caregivers only]** What has been your role as a family member/closest environment since diagnosis? What has changed in your relationship with the patient?
2. **Life before CKD:** What was your life like before you were diagnosed with CKD? What things have changed the most? What do you miss?
   1. Since you were diagnosed with how your lifestyle has changed? How do you feel about that? What could help you?
      1. How has the disease changed your relationship with your job?
      2. With your family and environment?
      3. And his personal life in general?
   2. At the beginning of the interview you explained what a day in your life is like. How would this change if life were like it used to be?
   3. Could you specify how you think the disease changed your life? [Moderator: Inquire into topics such as new interpretations of reality, change in the person's values, etc.]

| **Area of exploration** | **5. PATIENT'S EMOTIONAL AND CARE PROCESS (30 min)** |
| --- | --- |
| **Objectives and information** | - **Understand the patient's *Journey*** - **Identify unmet needs** - **Understand the perception of the different treatments the patient has gone through** |
| **Ethnographic explanation** | *I would now like to delve into the process that has been followed since the risk factors began to this day...* |

Next we will talk about the video/audio that you send before the interview to explain how the evolution of the disease has been and the different phases through which it has gone.

***
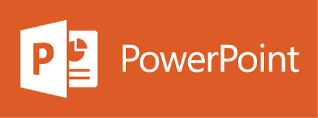
***

***EXERCISE #2: PATIENT JOURNEY***

Digital materials:

- Patient journey
- Cards depicting patient journey
- Cards depicting emotions

**Moderator:** For this section you must prepare a simple journey according to what the patient has explained in the video/pre-interview and prepare the questions in this section according to the gaps of information you identify.

The main stages to be explored are:

1. Life without disease
2. Early symptoms
3. Diagnosis
4. Follow-up and symptomatology
5. Current Life
6. Hyperkalaemia events (if applicable)
7. Dialysis (if applicable)


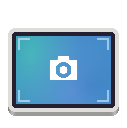


*Once the journey is created, take a screenshot of the result*.

**For each stage:**

- - Could you explain to me what this stage was like? How did it feel?
  - What was the **impact** of CKD at the moment?
  - And their **quality of life** was affected? In what sense?
  - Could you explain to me some **experience** that exemplifies how I lived this stage?
  - Of the **keywords** we have shown above [Display Screen Moderator], which would best describe this stage?
  - With which **healthcare professionals**did you interact during this stage? What was the relationship like? What did they bring you? How could they have brought you more value? [Moderator to deepen the role of MAP, nephrologist, cardiologist and nurse in the different phases].
  - Were you taking any **treatment**? Remember which one? Did you ever stop the treatment? What could have helped you?

**Specific questions for each phase/stage** [Moderator, adapt as appropriate]

1. **Life without Disease:** What was your life like before the disease? If you look back, what do you miss the most? What would it change?
2. **Previous Illnesses**: What diseases diagnosed you? Who?
3. Have you been warned of the risk of developing CKD?
4. What did they explain to you?
5. Did you recommend making lifestyle changes? [Moderator, ask for exercise, diet, tobacco or alcohol consumption...]
6. Were you given any preventive treatment?
7. **Early symptoms** [Moderator, investigating differences in patients with and without heart failure]:
8. What changes did you experience that made you think something was wrong? For how long?
9. What did you think might be going on?
10. **Diagnosis** [Moderator, investigating differences in patients with and without heart failure]:
11. Do you remember being told?
12. What tests did they do? Was the process heavy?
13. What doubts did you have?
14. What did you think was going to happen then?
15. Did you search the internet for information?
16. Did you contact any patient associations?
17. Have you met anyone with this condition during the process? How has it affected you?
18. **Monitoring:**

**First treatment:**

1. Do you remember the name of the treatment?
2. How should I take that first drug?
3. Did you have a routine to do it?
4. Did you ever stop taking the treatment?

**Follow-up visits:**

1. What are the visit routines like? How is the process?
2. How has it changed with COVID?
3. Did you have trouble making them? How do you currently feel when you have to go to the doctor?
4. What are the main healthcare professionalsyou see? What is your opinion of each of them?
5. How do you emotionally live the process of follow-up visits?

**Evolution of the disease:**

1. How did you find out that the disease was changing? Was there any significant event?

**Change of treatment** [Moderator note the treatment topology you mention, try to find out what it is if the patient does not mention it].

1. What did they explain to you when they switched you?
2. If you've done it several times, do you remember all the treatments you've had for CKD? [Moderator note and ask for each:]
3. How did he take it?
4. What was the experience of taking it like?
5. **Symptoms:**
   1. What are the main symptoms of the disease?
   2. How do you usually handle them? Are they easy to identify?
   3. How do they impact your quality of life?
   4. What do they deprive you of? What have you stopped doing?

1. **Hyperkalaemia:**
   1. On what occasions have you had elevated serum potassium levels? What were these episodes like?
   2. How often does it happen to you?
   3. How do you usually handle these situations?
   4. What measures does it take to prevent this from happening?
   5. How does all this impact your quality of life?
2. **Transplant**
   1. When did you find that you were going to receive a transplant?
   2. How did you feel emotionally?
   3. Could you describe to me what the process was like?
   4. Did they give you any kind of written information?

*If you have not yet been transplanted:*

- 1. Have you been told about the transplant?
  2. What is your opinion?

1. **Dialysis** [If applicable]
   1. How does the dialysis process live?
   2. How do you feel emotionally?
   3. How does it impact your quality of life?
2. **Needs.** Now that we have reviewed the experience of [patient name], do you think there is anything that could have been done better? Anything that would have helped him in particular?
3. **Experience with the quality of life questionnaire:** before the interview you had to answer a questionnaire about your quality of life.
   1. What things do you think should be included in this questionnaire?
   2. What things should your nephrologist know to make decisions?

This would conclude the interview. Thank you very much for your time and participation. Do you have any comments or questions you would like to share?

**FINISH RECORDING**

**DIGITAL JOURNAL (5 days)**

Scanning topics per day:

- D1: Everyday life
- D2: Emotions
- D3: Experience with the disease
- D4: Information search
- D5: Home tour (after interview)

| **Area of exploration** | **Day 1: Presentation** |
| --- | --- |

Good morning, [name of patient or caregiver]. My name is [name of the investigator who will conduct the interview] and I will be the one who interviewed you on the day [interview date]. As you have agreed, today we will start a digital newspaper. From today until the [day after the interview], we will send you simple questions about your day to day that you can answer when it suits you best and in the way you want (with audio, text or images). I remind you that this information is absolutely confidential and will only be used for the purposes of this study. Feel free to contact me if you have any questions or comments.

| **Area of exploration** | **Day 1: Everyday life** |
| --- | --- |
| **Goals** | - **Build a relationship with the patient** - **Understand your daily habits and routines** - **Knowing some personality traits** |

Today we want to know what a day in your life is like. We will ask you to answer these questions throughout the day.

1. Tell us about the activities you do during the day
2. Tell us about the people you've talked to during the day

We invite you to send photos and videos, if you wish.

[Reminder message, if applicable]

Remember, this is your diary. You can tell us what happens to you today. Some guiding questions:

1. What activities have you done during the day? Do you have a habit of taking care of yourself or relaxing?
2. Who did you talk to today? What do they mean to you?
3. What have you shared with those around you?

[Reminder message, if applicable]

Before going to bed, could you answer these questions? Remember that you can send messages, voice notes, or video.

1. What was your favorite moment of the day and why?
2. What was your least favorite moment and why?
3. If it were a weekend day... what activities would you tell us about change?

Good night! Tomorrow we continue.

| **Area of exploration** | **Day 2: Emotions** |
| --- | --- |
| **Goals** | - **Build a relationship with the patient** - **Know the emotions that prevail during their day to day** |

Good morning!

Thinking about how you feel about your illness and all the changes it brings to your day-to-day life, think about the positive and negative emotions you feel.

1. Send us an image that represents each of the emotions you feel.

[Reminder message, if applicable]

Remember to tell us about the emotions you feel throughout the day. We invite you to send us an image that represents the emotions you feel.

[Reminder message, if applicable]

Sometimes it's good to share with loved ones. If you were with a friend or close friend, what would you say about the changes you are currently experiencing? Would you ask them for something?

Good night! Tomorrow we continue.

| **Area of exploration** | **Day 3: Life with the disease** |
| --- | --- |
| **Goals** | - **Build a relationship with the patient** - **Know your emotional and care journey with ERC** |

Good morning,

Today I would like you to share what your experience with your disease has been like to this day. We'll keep talking about it during the interview.

1. Could you make a video of your experience?
2. Have there been any different times since you were diagnosed to this day? What are those moments?
3. If you had to choose the most difficult time? What would it be?
4. And the time with your most encouraging illness? What would it be? Why is that?

[Reminder message, if applicable]

Remember to share the most important moments of your life with the disease. You can share a video or an audio message. If you want, you can also send text messages.

Good night! Tomorrow we continue.

| **Area of exploration** | **Day 4: Search for information Social networks** |
| --- | --- |
| **Goals** | - **Understand the channels of information you regularly use and your social media activity (if applicable)** |

Good morning! A few questions to start the day:

1. When you're looking for information about your disease or how to improve your quality of life... Where do you consult? Where are you looking for information? Who are you asking?
2. Send us a photo or a screenshot of some information you liked and helped you a lot (it could be from a book, the internet, something your doctor gave you, or something you saw on social media).
3. Do you follow the advice of anyone or any particular organization? Can you send us a photo or screenshot of any content or information you like about this person or organization?

[Reminder message, if applicable]

Remember to send us content that has helped you to learn more about your condition or to improve your quality of life. It can be anything: an image, a video, an advertisement, a message, a profile or the page that follows.

[Reminder message, if applicable]

Before you go to bed, remember to send us some information that has helped you to better manage your illness and/or your quality of life. It can be anything, anything you've found or shared with you, or something that has inspired you.

Good night!

| **Area of exploration** | **Day 5: Home Tour (post-interview)** |
| --- | --- |
| **Goals** | - **To complement and contrast the information obtained during the interview with the patient's environment.** - **Understand the general context in which you live (routines, rituals and concessions to the ERC)** |

Last day! Thank you for your time during the interview. Today I would like to talk about the rituals or activities that you perform at home and that help you live with the disease:

1. Do you have a favorite corner of your home where you relax or do an activity that distracts you? (e.g. reading, exercising, meditating, etc.) What is your favorite place and activity?
2. Where do you keep information about your treatments? Does having it handy help you? Do you have materials or books that help or have helped you with your illness? Can you send me an example photo?
3. Have you had to make any home refurbishments or adaptations to help you live better with the disease? Which one?

[Reminder message, if applicable]

Remember to share your favorite corners of your home and what has helped you live better with the disease. It can be anything: an image, a message, an audio or a video.

[Reminder message, if applicable]

Before you go to bed, remember our messages from today. Thank you very much for your participation and good night.
